# Synthesis of *ent*-Kaurane Diterpene Monoglycosides

**DOI:** 10.3390/molecules16108402

**Published:** 2011-10-03

**Authors:** Venkata Sai Prakash Chaturvedula, Josef Klucik, Mani Upreti, Indra Prakash

**Affiliations:** Organic Chemistry Department, Global Research and Development, The Coca-Cola Company, One Coca-Cola Plaza, Atlanta, GA 30313, USA

**Keywords:** *ent*-kaurane diterpene glycosides, nor *ent*-kaurane glycoside, steviol, isosteviol, synthesis, structure characterization, spectral data

## Abstract

Synthesis of two *ent*-kaurane diterpene glycosides, steviol 19-O-β-D-glucopyranosiduronic acid (steviol glucuronide, **5** ), and 13-hydroxy *ent*-kaur-16-en-19-oic acid-β-D-glucopyranosyl ester (**7**) has been achieved from a common starting material, steviol, using phase transfer catalyst. Also, synthesis of an additional 17-nor-*ent*-kaurane glycoside, namely 13-methyl-16-oxo-17-nor-*ent*-kauran-19-oic acid-β-D-glucopyranosyl ester (**10**) was performed using the starting material isosteviol and similar synthetic methodology. Synthesis of all three steviol glycosides was performed using straightforward chemistry and their structures were characterized on the basis of 1D and 2D NMR as well as mass spectral (MS) data.

## 1. Introduction

The major constituents isolated from the leaves of *Stevia rebaudiana* Bertoni (family: Asteraceae) are the potently sweet diterpenoid glycosides stevioside, and rebaudioside A. These compounds which are known as Stevia sweeteners are glycosides of the diterpene steviol, *ent*-13-hydroxykaur-16-en-19-oic acid [1]. Stevioside tastes about 150-250 times sweeter than sucrose, whereas rebaudioside A tastes about 200-300 times sweeter than sucrose; both are non-caloric. In some parts of the world, including Japan, South Korea, Israel, Mexico, Paraguay, Brazil, Argentina, and Switzerland, these steviol glycosides are used to sweeten food products and beverages. As a part of our continuing research to discover natural sweeteners, we have reported the isolation of several glycosides from the commercial extract of *S. rebaudiana* [2,3,4,5,6,7,8]. Apart from isolating novel compounds from *S. rebaudiana* and utilizing them as possible natural sweeteners or sweetness enhancers, we are also engaged in understanding the physicochemical profiles of steviol glycosides in various systems of interest and their metabolites, as well as their characterization [9]. Though many steviol glycosides have been reported in the literature, focused synthetic studies were not carried out. In this article, we present the synthesis of three *ent*-kaurane diterpene glycosides possessing steviol and isosteviol skeletons, and the characterization of their structures based on extensive NMR and mass spectroscopic data.

## 2. Results and Discussion

### 2.1. Chemistry

**Scheme 1:**
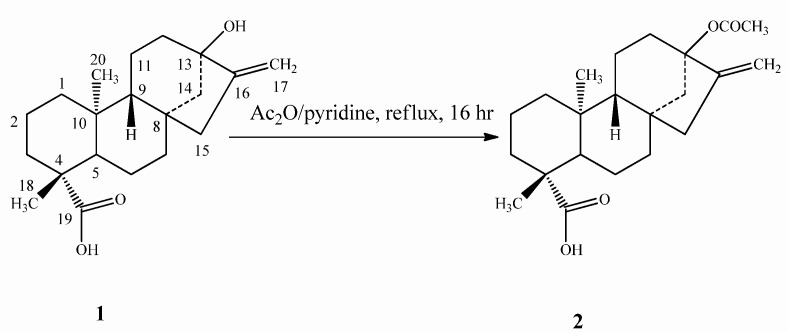
Synthesis of steviol acetate (**2**).

The two compounds **5** and **7** were synthesized via the common intermediate 13-acetyloxy-*ent*-kaur-16-en-19-oic acid (steviol acetate, **2**), prepared by acetylation of steviol (**1**) with Ac_2_O and pyridine (Scheme 1).

**Scheme 2:**
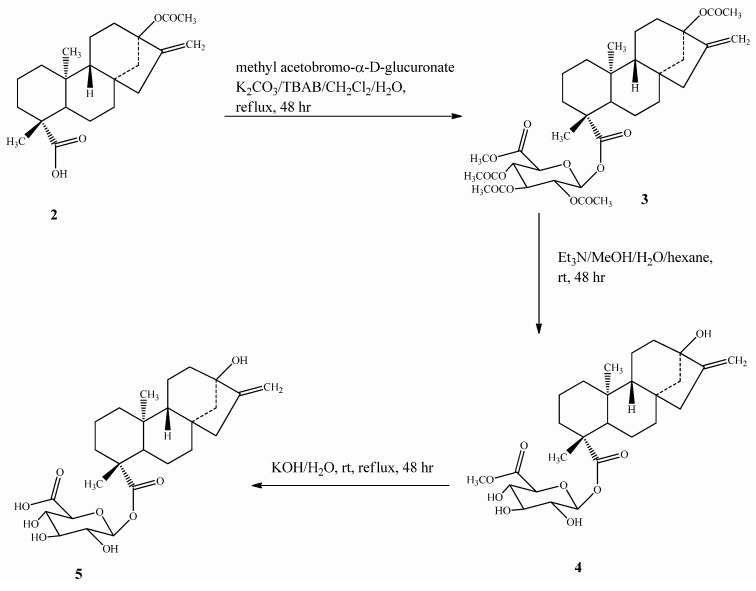
Synthesis of steviol glucuronide (**5**).

**Scheme 3:**
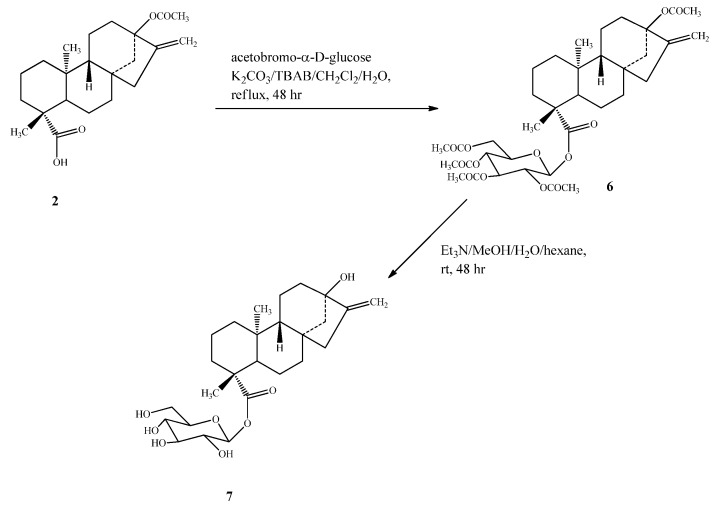
Synthesis of 13-hydroxy *ent*-kaur-16-en-19-oic acid-β-D-glucopyranosyl ester (**7**).

Compound **5** was prepared by esterification of **2** with bromo-2,3,4-tri-*O*-acetyl-α-D-glucopyran-uronic acid methyl ester (methyl acetobromo-α-D-glucuronate) in the presence of the phase transfer catalyst tetrabutylammonium bromide (TBAB) to yield **3**, which upon deacetylation using Et_3_N, followed by alkaline hydrolysis, furnished the final steviol glucuronide (**5**) (Scheme 2). Compound **7** was synthesized from **2** using the same esterification procedure as mentioned for **5** with 2,3,4,6-tetra-*O*-acetyl-α-D-glucopyranosyl bromide**(**acetobromo-α-D-glucose**)** to furnish intermediate **6**, which on deacetylation using Et_3_N afforded the final compound **7** **(**Scheme 3).

**Scheme 4:**
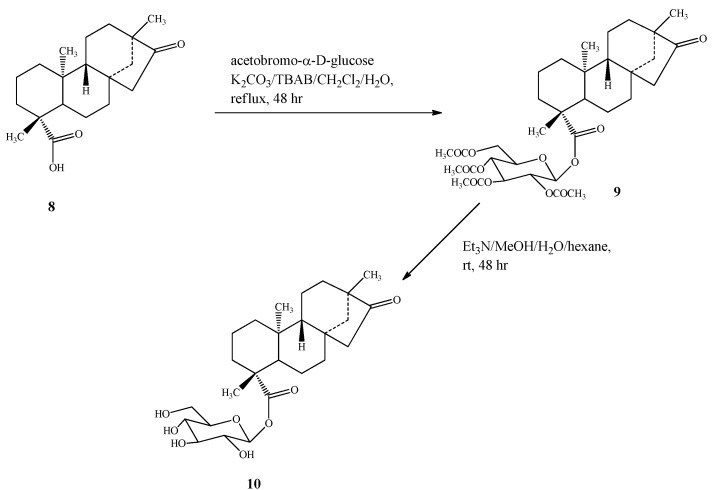
Synthesis of 13-methyl-16-oxo-17-nor-*ent*-kauran-19-oic acid-β-D-gluco-pyranosyl ester (**10**).

Compound **10** was prepared by esterification of isosteviol (**8**) with acetobromo-α-D-glucose in the presence of phase transfer catalyst as described for **7** , producing intermediate **9**, which on deacetylation with Et_3_N as reported above yielded compound **10** (Scheme 4).

### 2.2. Spectroscopy

The structural characterization of **5** , **7** and **10** was performed on the basis of one dimensional (^1^H, ^13^C), two-dimensional (^1^H-^1^H COSY, ^1^H-^13^C HMQC, ^1^H-^13^C HMBC) NMR and mass spectral data, as well as in comparison with literature values [3,10]. The attachment of sugars at the C-19 position of the steviol skeleton in **5** and **7** , and the isosteviol skeleton in **10** was characterized by the key HMBC correlation of the anomeric protons of the respective sugar moieties with the C-19 carbonyl group. The ^1^H- and ^13^C-NMR values for all the protons and carbons were assigned on the basis of COSY, HMQC and HMBC correlations and are given in Table 1 and Table 2. The anomeric protons in all three glycosides 5, **7** and **10** were observed as doublets at δ 5.44 (d, 7.8 Hz), 5.41 (d, 8.2 Hz) and 5.38 (d, 8.2 Hz) respectively, suggesting their β-configuration similar to that of the steviol glycosides isolated from *S. rebaudiana* [2,3,4,5,6,7,8]. This suggested that even though esterification was performed using their corresponding α-derivatives; the configuration of the final products **5** , **7** , and **10** was observed to correspond to their β-derivatives; an identical phenomenon was reported earlier for glycosylation of triterpene acids under phase transfer catalytic conditions [11].

**Table 1:** ^1^H-NMR chemical shift values for compounds **5** , **7** and **10** * recorded in CD_3_OD ^a^.

Position	5	7	10
1	0.86 (m, 1H), 1.86 (m, 1H)	0.86 (m, 1H), 1.88 (m, 1H)	0.96 (m, 1H), 1.68 (m, 1H)
2	1.39 (m, 1H), 1.90 (m, 1H)	1.43 (m, 1H),1.93 (m, 1H)	1.36 (m, 1H), 1.92 (m, 1H)
3	1.02 (m, 1H), 2.26 (d, 11.9, 1H)	1.06 (m, 1H), 2.19 (d, 12.4, 1H)	1.05 (m, 1H), 2.16 (d, 13.2, 1H)
5	1.08 (m, 1H)	1.12 (m, 1H)	1.20 (m, 1H)
6	1.82 (m, 1H), 1.93 (m, 1H)	1.85 (m, 1H), 1.96 (m, 1H)	1.86 (m, 2H)
7	1.45 (m, 1H), 1.56 (m, 1H)	1.44 (m, 1H), 1.54 (m, 1H)	1.47 (m, 1H), 1.66 (m, 1H)
9	0.88 (m, 1H)	0.96 (m, 1H)	1.24 (m, 1H)
11	1.60 (m, 1H), 1.79 (m, 1H)	1.61 (m, 1H), 1.77 (m, 1H)	1.25 (m, 1H), 1.70 (m, 1H)
12	1.53 (m, 1H), 1.87 (m, 1H)	1.46 (m, 1H), 1.74 (m, 1H)	1.42 (m, 1H), 1.56 (m, 1H)
14	1.58 (m, 1H), 2.23 (d, 12.1, 1H)	1.28 (m, 1H), 2.11 (m, 1H)	1.44 (m, 1H), 1.58 (m, 1H)
15	2.02 (m, 1H), 2.16 (d, *J =* 17.4, 1H)	2.06 (m, 1H), 2.17 (m, 1H)	1.80 (m, 1H), 2.65 (dd, 3.1, 17.2, 1H)
17	4.59 (s, 1H), 4.78 (br s, 1H)	4.77 (br s, 1H), 4.93 (s, 1H)	
18	1.23 (s, 3H)	1.20 (s, 3H)	1.24 (s, 3H)
20	0.97 (s, 3H)	0.98 (s, 3H)	0.93 (s, 3H)
1′	5.44 (d, 7.8, 1H)	5.41 (d, *J =* 8.2 Hz, 1H)	5.38 (d, 8.2, 1H)
2′	3.60 (dd, 8.2, 9.1, 1H)	3.35 (dd, 7.1, 7.6, 1H)	3.32 (dd, 7.2, 7.8, 1H)
3′	3.42 (dd, 8.1, 8.9, 1H)	3.44 (dd, 8.3, 9.1, 1H)	3.43 (dd, 8.1, 9.1, 1H)
4′	3.49 (dd, 8.1, 9.2, 1H)	3.34 (dd, 8.2, 9.4, 1H)	3.34 (dd, 8.1, 9.1, 1H)
5′	3.72 (d, 8.2, 1H)	3.39 (ddd, 8.1, 2.1, 7.4, 1H)	3.37 (ddd, 8.2, 1.9, 7.2, 1H)
6′		3.66 (dd, 2.1, 12.1, 1H), 3.81 (dd, 4.2, 12.1, 1H)	3.68 (dd, 1.9, 12.1, 1H), 3.82 (dd, 3.9, 12.1, 1H)

^a^ assignments made on the basis of COSY, HMQC and HMBC correlations; ^b^ Chemical shift values are in δ (ppm); ^c^ Coupling constants are in Hz; * δ_H_ 0.80 (s, 3H): CH_3_-13 for compound **10** .

**Table 2:** ^13^C-NMR chemical shift values for **5** , **7** and **10** * recorded in CD_3_OD ^a^.

Position	1	2	3
1	42.0	41.5	39.5
2	20.3	20.2	19.1
3	39.2	38.6	38.0
4	45.2	45.1	43.9
5	58.7	58.5	57.6
6	23.1	22.8	20.7
7	42.8	42.4	41.4
8	42.9	43.0	48.4
9	55.5	55.0	53.9
10	40.9	40.5	37.6
11	21.5	21.3	21.1
12	40.7	40.4	38.6
13	81.0	88.5	39.1
14	47.4	47.2	54.6
15	48.8	48.6	48.3
16	157.2	157.0	223.8
17	103.5	103.4	
18	29.1	29.1	27.6
19	178.2	178.0	176.8
20	16.5	16.3	19.1
1′	95.6	95.6	95.5
2′	78.7	74.0	73.8
3′	74.0	77.7	77.3
4′	73.6	71.0	70.8
5′	77.7	77.8	77.6
6′	177.5	61.2	61.1

^a^ assignments made on the basis of HMQC and HMBC correlations; ^b^ Chemical shift values are in δ (ppm); * δ_C_ 12.8: CH_3_-13 for compound **10** .

## 3. Experimental

### 3.1. General

Melting points were measured using a SRS Optimelt MPA 100 instrument and are uncorrected. Optical rotations were recorded using a Rudolph Autopol V at 25 °C and IR spectral data was acquired using a Perkin Elmer 400 Fourier Transform Infrared (FT-IR) spectrometer equipped with a universal attenuated total reflectance (UATR) polarization accessory. NMR spectra were acquired on Bruker Avance DRX 500 MHz using standard pulse sequences. Chemical shifts are given in δ (ppm), and coupling constants are reported in Hz. HRMS and MS/MS data were generated with a Waters Premier Quadrupole Time-of-Flight (Q-TOF) mass spectrometer equipped with an electrospray ionization source operated in the positive-ion mode and ThermoFisher Discovery OrbiTrap in the positive mode of electrospray. Samples were diluted with water: acetonitrile (1:1) containing 0.1% formic acid and introduced via infusion using the onboard syringe pump. HPLC was performed on an Agilent 1100 system using a Phenomenex Prodigy ODS (3) column (250 × 21.2 mm, 5 μm) or a Beckman Gold system with a Zorbax amino (150 × 4.6 mm, 5 μm) column.

### 3.2. Isolation

Purification of compound **5** was carried out using the Beckman HPLC system using an isocratic solvent method; UV Detection: 210 nm; Mobile Phase A: 75% CH_3_CN/25% H_2_O/0.05% AcOH; Mobile Phase B: 100% CH_3_CN/0.05% AcOH; Flow Rate: 1.0 mL/min; Gradient ratio: Mobile Phase A/B (60:40), by collecting the peak eluting at *t_R_* 1.33 min. Compound **7** was purified by using the Agilent HPLC 1100 system. UV Detection: 210 nm; Mobile Phase A: H_2_O (0.02% AcOH, 0.08% NH_4_OAc); Mobile Phase B: CH_3_CN; using gradient [25% B for 8.5 min, 25 to 29% B over 1.5 min, 29 to 30% B over 5.5 min, 30 to 34% B over 4.0 min, 34% B for 6 min, 34 to 52% B over 2.0 min, 52% B for 3.0 min, 52 to 70% B over 1.0 min, 70% B for 5.5 min] at 5 mL/min flow rate. The peak eluting at *t_R_* 28.3 min corresponds to **7**. Compound **10** was also purified using the Agilent HPLC 1100 system. UV Detection: 226 nm; Mobile Phase A: H_2_O (0.1% TFA); Mobile Phase B: CH_3_CN; gradient increased from 95:5 (A:B) to 0:100 (A:B) over 30 min; Flow Rate: 5.0 mL/min. The peak eluting at *t_R_* 18.2 min furnished **10**.

*Steviol acetate* (**2**): Steviol acetate (**2**) was prepared from steviol (**1**) as reported in the literature and characterized based on the ^1^H-NMR and mass spectral data and comparison with the spectral data reported in the literature [12]. ^1^H-NMR (500 MHz, CDCl_3_): δ 4.92 (s, 1H, 17-H), 4.86 (s, 1H, 17-H), 2.03 (s, 3H, OCOCH_3_); HRMS (M+Na)^+^*m/z* 383.2198 (calcd. for C_22_H_32_O_4_Na: 383.2208).

*Steviol glucuronide* (**5**): Compound **2** (0.633 g, 1.76 mmol) was dissolved in CH_2_Cl_2_ (25 mL) and distilled water (3 mL). TBAB (10 mg), K_2_CO_3_ (0.62 g, 4.50 mmol), and methyl acetobromo-α-D-glucuronate (0.80 g, 2.02 mmol) were added at room temperature. The reaction mixture was refluxed for 48 h, cooled to room temperature and the layers were separated. The aqueous layer was extracted with additional amounts of CH_2_Cl_2_ (2 × 20 mL) and the combined organic layer was washed with water (2 × 30 mL) and brine (40 mL). Concentration of the CH_2_Cl_2_ layer under vacuum furnished a residue (1.05 g), which showed a peak at *m/z* 677 in its mass spectrum corresponding to the (M+H)^+^ ion of **3**. Compound **3** (1.00 g, 14.7 mmol) was dissolved in 10% solution of Et_3_N in MeOH-H_2_O-hexane (10:2:1, 23 mL) and stirred at room temperature for 48 h. The MeOH and hexane were evaporated under vacuum and the resulting product (0.73 g) was identified as **4** on the basis of its mass spectrum, which showed a peak at *m/z* 509 corresponding to its (M+H)^+^ ion. Compound **4** (0.70 g) was dissolved in H_2_O (20 mL) and KOH (0.16 g, 0.28 mmol) was added. The mixture was stirred at room temperature for 48 h and the mixture was subjected to evaporation under reduced pressure. Purification of the residue obtained after evaporation was performed using HPLC furnished 420 mg of **5**. White powder, mp 198.7 °C, [α]_D_^25^ −70.6 (*c* 1.0, MeOH); IR ν_max_: 3303, 2937, 1723, 1599, 1053, 970 cm^−1^; ^1^H-NMR (500 MHz, CD_3_OD, δ ppm) and ^13^C-NMR (125 MHz, CD_3_OD, δ ppm) spectroscopic data see Table 1 and Table 2; HRMS (M+NH_4_)^+^*m/z* 512.2852 (calcd. for C_26_H_42_O_9_N: 512.2860); (M+Na)^+^*m/z* 517.2405 (calcd. for C_26_H_38_O_9_Na: 517.2414).

*13-Hydroxy-ent-kaur-16-en-19-oic acid-β- D-glucopyranosyl ester* (**7**): To a solution of **2** (2.00 g, 5.56 mmol) in CH_2_Cl_2_ (35 mL) and distilled water (4 mL); TBAB (30 mg), K_2_CO_3_ (1.94 g, 14.06 mmol), and acetobromo-α-D-glucose (2.88 g, **7** mmol) were added at room temperature. The reaction mixture was refluxed for 48 h, cooled to room temperature and the layers were separated. The aqueous layer was extracted with additional CH_2_Cl_2_ (2 × 50 mL) and the combined organic layer was washed with water (2 × 60 mL) and brine (100 mL). Concentration of the CH_2_Cl_2_ layer under vacuum furnished a residue (2.23 g) which was identified as **6** on the basis of its EIMS spectrum which showed a peak at *m/z* 691 corresponding to its (M+H)^+^ ion. Compound **6** (2.00 g, 2.90 mmol) was dissolved in 10% solution of Et_3_N in MeOH-H_2_O-hexane (10:2:1, 45 mL) and stirred at room temperature for 48 h. The residue resulting after evaporation of the MeOH and hexane was purified by HPLC to furnish 850 mg of **7**. White powder, [α]_D_^25^ −62.4 (*c* 1.0, EtOH); IR ν_max_: 3350, 2929, 1725, 1033, 890 cm^−1^; ^1^H-NMR (500 MHz, CD_3_OD, δ ppm) and ^13^C-NMR (125 MHz, CD_3_OD, δ ppm) spectroscopic data see Table 1 and Table 2; HRMS (M+Na)^+^*m/z* 503.2608 (calcd. for C_26_H_40_O_8_Na: 503.2621).

*13-Methyl-16-oxo-17-nor-ent-kauran-19-oic acid-β- D-glucopyranosyl ester* (**10**): To a solution of **8** (0.70 g, 2.20 mmol) in CH_2_Cl_2_ (15 mL) and distilled water (4 mL) was added TBAB (15 mg), K_2_CO_3_ (1.00 g, 7.25 mmol), and acetobromo-α-D-glucose (1.25 g, 3.04 mmol). The reaction mixture was refluxed for 48 h, cooled to room temperature and worked-up as described above to furnish a residue (1.20 g) which showed a peak at *m/z* 649 in its EIMS spectrum corresponding to the (M+H)^+^ ion of **9**. Compound **9** (1.00 g, 1.54 mmol) was deacetylated as described above and upon usual work-up afforded a residue which was purified by HPLC to yield 500 mg of **10**. White powder, mp 172.5 °C, [α]_D_^25^ −55.8 (*c* 1.0, EtOH); IR ν_max_: 3352, 2928, 1726, 1034, 891 cm^−1^; ^1^H-NMR (500 MHz, CD_3_OD, δ ppm) and ^13^C-NMR (125 MHz, CD_3_OD, δ ppm) spectroscopic data see Table 1 and Table 2; HRMS (M+Na)^+^*m/z* 503.2611 (calcd. for C_26_H_40_O_8_Na: 503.2621).

## 4. Conclusions

In conclusion, three *ent*-kaurane diterpene glycosides **5**, **7** and **10** were synthesized from the natural products steviol and isosteviol through simplified procedures. To the best of our knowledge, this is the first report of the synthesis of C-19 glycosidic linkages on the steviol and istosteviol skeletons. The structures of all the synthesized compounds were characterized on the basis of NMR (1D and 2D) and mass spectral data, as well as in comparison with the data reported in the literature.
